# Using Continuous Passive Assessment Technology to Describe Health and Behavior Patterns Preceding and Following a Cancer Diagnosis in Older Adults: Proof-of-Concept Case Series Study

**DOI:** 10.2196/45693

**Published:** 2023-08-10

**Authors:** Chao-Yi Wu, Deanne Tibbitts, Zachary Beattie, Hiroko Dodge, Jackilen Shannon, Jeffrey Kaye, Kerri Winters-Stone

**Affiliations:** 1 Department of Neurology Oregon Health & Science University Portland, OR United States; 2 Department of Neurology Massachusetts General Hospital Harvard Medical School Charlestown, MA United States; 3 Division of Oncological Sciences Oregon Health & Science University Portland, OR United States; 4 Knight Cancer Institute Oregon Health & Science University Portland, OR United States

**Keywords:** sensor, quality of life, physical activity, medication, monitoring, function, mobile phone

## Abstract

**Background:**

Describing changes in health and behavior that precede and follow a sentinel health event, such as a cancer diagnosis, is challenging because of the lack of longitudinal, objective measurements that are collected frequently enough to capture varying trajectories of change leading up to and following the event. A continuous passive assessment system that continuously monitors older adults’ physical activity, weight, medication-taking behavior, pain, health events, and mood could enable the identification of more specific health and behavior patterns leading up to a cancer diagnosis and whether and how patterns change thereafter.

**Objective:**

In this study, we conducted a proof-of-concept retrospective analysis, in which we identified new cancer diagnoses in older adults and compared trajectories of change in health and behaviors before and after cancer diagnosis.

**Methods:**

Participants were 10 older adults (mean age 71.8, SD 4.9 years; 3/10, 30% female) with various self-reported cancer types from a larger prospective cohort study of older adults. A technology-agnostic assessment platform using multiple devices provided continuous data on daily physical activity via wearable sensors (actigraphy); weight via a Wi-Fi–enabled digital scale; daily medication-taking behavior using electronic Bluetooth-enabled pillboxes; and weekly pain, health events, and mood with online, self-report surveys.

**Results:**

Longitudinal linear mixed-effects models revealed significant differences in the pre- and postcancer trajectories of step counts (*P*<.001), step count variability (*P*=.004), weight (*P*<.001), pain severity (*P*<.001), hospitalization or emergency room visits (*P*=.03), days away from home overnight (*P*=.01), and the number of pillbox door openings (*P*<.001). Over the year preceding a cancer diagnosis, there were gradual reductions in step counts and weight and gradual increases in pain severity, step count variability, hospitalization or emergency room visits, and days away from home overnight compared with 1 year after the cancer diagnosis. Across the year after the cancer diagnosis, there was a gradual increase in the number of pillbox door openings compared with 1 year before the cancer diagnosis. There was no significant trajectory change from the pre– to post–cancer diagnosis period in terms of low mood (*P*=.60) and loneliness (*P*=.22).

**Conclusions:**

A home-based, technology-agnostic, and multidomain assessment platform could provide a unique approach to monitoring different types of behavior and health markers in parallel before and after a life-changing health event. Continuous passive monitoring that is ecologically valid, less prone to bias, and limits participant burden could greatly enhance research that aims to improve early detection efforts, clinical care, and outcomes for people with cancer.

## Introduction

### Background

Owing to improved survival rates, cancer is now increasingly considered a chronic illness that an individual will manage for the rest of their lives. Throughout survivorship, one could be faced with ongoing systemic treatments, the management of complex and persistent symptoms and side effects, long-term and late effects that manifest after treatment ends, possible recurrence, and the diagnosis of a second primary cancer. Anticipating and managing the health of cancer survivors could be better achieved if there is a better understanding and system of surveillance for how various dimensions of health and behavior change before and throughout survivorship. Current approaches are mostly limited to conventional measurement techniques that rely on self-reported information often collected at a health care visit or other episodic intervals, which can be subject to memory recall lapses and patient bias [1]. In addition, provider-based assessments during a health care visit often do not reflect the patient’s health in their home, missing daily life events occurring between visits when health may be vacillating or declining. In general, measures episodically reported during clinical visits provide low-frequency information covering a limited number of health domains that are not ecologically valid because they fail to capture everyday behaviors. With typical self-report measures, information about how behaviors and health markers change over time and before and after a specific event lack the granularity that could improve early detection and prediction of health outcomes, as well as the efficacy of cancer treatment.

Rapidly emerging digital technologies (eg, wearable devices, unobtrusive environment sensors, and passive data collection applications) provide an opportunity to track the patterns and trajectories of health and behaviors across survivorship. Using home-based digital technology to measure key health domains (eg, mobility, cognition and behavioral health, and socialization) could also provide a more informed and comprehensive understanding of the presentation and progression of health and behaviors as they occur in real life. Continuous passive monitoring overcomes the aforementioned limitations of infrequent, self-reported measures in longitudinal studies by providing an opportunity to follow multiple dimensions of health and behaviors across the entire cancer trajectory (ie, from the prediagnosis period through to the end of life). A technology-agnostic assessment platform can host various types of sensors, devices, or technologies, providing a flexible mechanism to deliver high-frequency and multidomain data in real time to effectively monitor individuals’ health changes or health events (eg, hospitalization and medication changes). Continuous passive assessment techniques have also gained popularity since the outbreak of the COVID-19 pandemic [2] as a way to follow patients without jeopardizing the health system’s capacity and health care workers’ safety [3]. Continuous passive assessment techniques could be useful in the context of cancer to monitor changes in health and behavior across survivorship. Clinicians have expressed a high level of acceptance toward using digital health data for planning cancer care [4], and cancer survivors have reported to be comfortable self-monitoring various health conditions after discharge from the hospital [5]. Passive assessments provide reliable complementary data compared with in-person collected data [6] and could be more ecologically valid when tracking cancer survivors’ health over time.

### Objective

The Collaborative Aging Research Using Technology (CART) initiative was a longitudinal observational study led by the Oregon Center for Aging & Technology to establish a passive monitoring assessment platform that could widely assess health and behaviors in the homes of diverse cohorts of older adults. Using the CART study cohort, we had the opportunity to explore a potential use case for a passive monitoring platform to track health and behaviors in individuals with cancer. We conducted a proof-of-concept retrospective analysis, in which we identified new cancer diagnoses in older adults in the CART study cohort and then compared trajectories of change in health and behaviors before and after cancer diagnosis. Health and behaviors were measured using the CART remote assessment platform, which continuously measures physical activity, body weight, medication-taking behavior, self-reported pain, health events (eg, hospitalization or emergency room [ER] visits), and mood (eg, low mood and loneliness). The ability of this approach to continuously track changes in health and behaviors before and throughout a major health event, such as a cancer diagnosis, demonstrates the feasibility of using this platform to improve early detection efforts, clinical care, and outcomes for people with cancer.

## Methods

### Overview

Secondary data for this analysis were obtained from the CART study, a longitudinal study examining aging, Alzheimer's disease, and related dementias in community-dwelling older adults [7,8]. Participants were recruited between 2018 and 2020. The participants were either living alone or with a cohabitant.

### Ethics Approval

These secondary data were part of the CART study for which the Oregon Health & Science University (IRB #17123) and Veterans Affairs Portland Health Care System (IRB #4089) institutional review boards approved the study protocol and consent forms.

### Informed Consent and Participation

All participants provided written informed consent before data collection. The consent form allows for secondary analyses by authorized investigators without additional consent.

The participants’ data were deidentified in this study. Participants were compensated US $59 per month for the duration of their participation in the CART study.

### Eligibility Criteria

Participants were included in the analysis if they (1) self-reported a new cancer diagnosis (ie, a type of cancer that had never been reported in either weekly online health questionnaires or annual assessments); (2) had data from 1 year before and 1 year following a self-reported cancer diagnosis; and (3) had all types of sensor data (wearable devices, electronic scales, electronic pillboxes, and online health and activity surveys).

### Measures

The technology-agnostic assessment platform used in the primary CART study (and this secondary study) comprised a wearable device, electronic scale, electronic pillbox, and online health and activity survey to observe longitudinal health and behavioral changes. In the primary CART study, devices, sensors, and technologies were selected based on their relevance to Alzheimer's disease and related dementias. These measures encompass various health domains, including physical activity, weight, medication-taking behaviors, pain, hospitalization or ER visits, and mood. The study technicians deployed the platform during an approximately 2-hour in-person home visit. The data were automatically transmitted to a central server cluster for annotation and storage. Detailed information regarding the platform installation and data collection is described elsewhere [7].

### Physical Activity

Upon study enrollment, the participants were asked to wear an actigraphy watch (Withings Steel watch). Physical activity was assessed using daily step counts [9] aggregated into weekly means and weekly variability. Step count variability was calculated using the coefficient of variation (weekly SD/weekly mean).

### Weight

Participants were provided with an electronic scale to measure their weight (Withings Body Cardio Scale). The participants were asked to weigh themselves daily using the scale. There were no guidelines or prompts regarding the specific time of a day that they should weigh themselves. For participants residing with another person, the average difference in weight of the 2 was used to distinguish the participant and cohabitant’s weight; this is feasible because of the large differences in weight between members of the dyad. For example, if the participant and cohabitant’s weights were 120 and 90 kg, respectively, 105 was used as a cutoff to differentiate the participant and cohabitant weights during the monitoring period.

### Medication-Taking Behavior

Participants were provided with an electronic wireless pillbox. The electronic pillbox is a 7-day pillbox that tracks medication-taking behavior by detecting the opening and closing of the 7 compartment doors [10,11]. Participants filled the box with at least 1 prescription medication. The participants used the pillbox according to their own medication-taking schedules. For participants with multiple pillboxes, data from all the pillboxes were combined and sorted by time for the analysis.

Two medication-taking behavior metrics were computed based on (1) the number of pillbox door openings on any given day and (2) the clock time of the first time any pillbox door was opened during each day. The pillbox door–opening metric was calculated as the average number of openings of the 7 compartment doors during the monitoring period. These data were used to track whether the participants had taken their pill. The pill-taking clock time metric was calculated as the average time of day the pillbox was first opened (in min from midnight) across the monitoring period and was presented as “clock time” in hours. To illustrate, if a participant opened the compartment doors at 7:30 AM and 8:45 PM, the clock times would be 7.5 and 20.75, respectively. The conversion of clock time to a real-number scale was intended for use in subsequent modeling. The clock time was used to track whether participants followed a consistent medication regimen. Variations in clock time was used to determine whether participants deviated from their medication regimen or forgot to take their medication.

### Weekly Pain, Health Events, and Mood

Every week, participants received online health surveys via email to self-report changes in pain severity, hospitalization or ER visits, days away from home overnight, medication changes, low mood, and loneliness [12-14]. Participants completed the survey using their own internet-connected device of choice (ie, tablet, smartphone, desktop, or laptop computer). The reasons for hospitalization or ER visits, days away from home overnight, and medication changes were recorded via self-reported free-text entry fields.

Pain severity was indicated by the number that best described pain on average in the last week from “No Pain (0)” to “Worst Imaginable (10).” Hospitalization or ER visits and days away from home overnight were identified using self-reported questions (yes or no). Medication changes were identified via a self-report question: “In the past week, have you had a change to any of your medications?” If participants had a change in their medications, they chose any of the following responses: new medication started (yes or no), stopped taking a medication (yes or no), dosage increased (yes or no), or dosage decreased (yes or no). Low mood (“Blueness”) was identified via a self-report question: “Have you felt downhearted or blue for 3 or more days in the past week (Yes/No)?” Loneliness was identified via a self-report question: “In the past week I felt lonely (Yes/No).” Questions regarding low mood and loneliness have been used in previous studies [14,15] and have shown their sensitivity to detect mood changes before and during the COVID-19 pandemic [15].

The severity of comorbidity was quantified using the modified Cumulative Illness Rating Scale-Geriatric [16]. The modified Cumulative Illness Rating Scale-Geriatric has 14 items; each item is rated on a 5-point Likert-type scale from 1 “None” to 5 “Extremely severe.” The total score ranges from 14 to 70, with higher scores indicating more severe comorbidities.

### Statistical Analysis

We extracted 104 weeks of data between 1 year before and 1 year after cancer diagnosis. Data collected after March 23, 2020 (when Oregon issued a stay-at-home order owing to the COVID-19 pandemic), were excluded because COVID-19–related restrictions may have changed participants’ behaviors [15].

We calculated the use rate of the device (actigraphy watch, scale, and electronic pillbox) by dividing the number of days with data by the number of monitored days. For the online health surveys, the response rate was calculated by dividing the number of weeks that had a survey response by the number of weeks that the survey was distributed. Days after death or the COVID-19 pandemic were excluded.

We first examined the time slope differences in continuous outcome variables (step counts, step count variability, weight, pillbox door opening, and pain severity) before and after cancer diagnosis using longitudinal linear mixed-effects models. We estimated the difference in slope between the 2 groups (pre- and postdiagnosis periods) using a group-by-time (wk) interaction term (the postdiagnosis period as the reference group). The interaction term coefficient indicates how much change occurred in the outcomes of interest 1 year before the diagnosis compared with 1 year after the diagnosis.

For dummy outcome variables (hospitalization or ER visits, days away from home overnight, blue mood, loneliness, and medication changes), we fitted generalized linear mixed models, with outcomes being the likelihood of the occurrence of low mood (eg, feeling blue or lonely) or health events (eg, hospitalization or ER visits). We estimated the difference in slope between the 2 groups (pre- and postdiagnosis periods) using a group-by-time (wk) interaction term (the postdiagnosis period as the reference group). The interaction term coefficient shows the change in the likelihood of reporting low mood or health events 1 year before diagnosis compared with 1 year after diagnosis.

## Results

### Overview

From the CART database, we found that 26 participants had reported a new cancer between 2018 and 2020, and 10 participants had data available between the pre– and post–cancer onset periods. A total of 10 older adults (mean age 71.8, SD 4.9 years; 3/10, 30% female) with various cancer diagnoses (esophageal, pancreatic, uterine, B-cell follicular lymphoma, multiple myeloma, prostate, squamous cell carcinoma, and basal cell carcinoma) were included in the analysis. One participant died during the monitoring period. Table 1 displays the average aggregated view of the daily data before and after diagnosis. Although an aggregated value provides a snapshot of information, it differs from high-frequency time-series data.

**Table 1:** Participant characteristics.

Characteristic			Age (years)		Sex		Comorbidity^a^		Education (years)		Step (counts/d)					Pain (severity/wk^b^)					Pillbox (openings/d)					Time of the day of pillbox opening					Weight (kg)		
											Before diagnosis		After diagnosis		Before diagnosis			After diagnosis		Before diagnosis			After diagnosis		Before diagnosis			After diagnosis		Before diagnosis			After diagnosis
**Individual participant identifiers and cancer types**																																	
	(A) Esophageal cancer	75.5		Male		27		10		3584.3		855.9		4.6			6.8		2.6			2.6		12:19 PM			12:34 PM		72.2			—^c^	
	(B) Pancreatic cancer	72.1		Female		24		18		1491		1799.7		4.4			5.9		—			3.6		—			11:43 AM		76.4			63.9	
	(C) Uterine cancer	76.7		Female		23		16		684.8		640.9		7.5			3.4		2.8			4		3:53 PM			2:37 PM		103.0			99.2	
	(D) B-cell follicular lymphoma	74.5		Male		28		18		1379.8		1148.8		4.0			4.1		2.3			2.2		5:36 AM			6:07 AM		105.2			—	
	(E) Multiple myeloma	65.9		Female		22		12		966.2		957.9		1.4			1.9		2.2			2.7		5:09 AM			7:50 AM		92.5			91.8	
	(F) Prostate cancer	71.6		Male		20		14		2214.9		1605.0		1.0			1.0		1.2			—		1:49 PM			—		124.2			123.5	
	(G) Squamous cell carcinoma	79.1		Male		29		18		733.2		1309.9		4.2			3.8		3.6			3.6		9:19 AM			9:11 AM		111.6			110.7	
	(H) Basal cell carcinoma	64.9		Male		20		20		9521.1		7335.5		1.1			1.1		10.9			10		12:21 PM			5:39 PM		86.5			87.9	
	(I) Basal cell carcinoma	66		Male		20		16		1899.2		1885.6		1.4			1.2		2			2.1		10:47 AM			11:14 AM		105.8			107.3	
	(J) Basal cell carcinoma	72		Male		20		15		497.9		599.7		3.8			5.0		—			—		—			—		104.7			100	
All participants, mean (SD)			71.8 (4.9)		N/A^d^		23.3 (3.6)		15.7 (3.1)		2397 (2643)		1914 (1950)		3.3 (2.1)			3.4 (2.1)		3.5 (3.1)			3.9 (2.6)		10:39 AM^e^			11:22 AM^e^		98.2 (16.1)			98.0 (17.8)

^a^Comorbidity was measured using the Cumulative Illness Rating Scale-Geriatric, with a higher score indicating worse health.

^b^Indicated by the number that best described pain on average in the last week from “No Pain (0)” to “Worst Imaginable (10).”

^c^Not available.

^d^N/A: not applicable.

^e^SD not available.

### Use and Response Rates

The use of the devices and response rates of the surveys are listed in Table 2. An average of 336 days of actigraphy data and 67 weeks of self-reported online survey data were collected from participants. The actigraphy watch’s use rate (number of days worn across the monitoring period) decreased by 13% from the pre– to post–cancer diagnosis period (from 69% to 56%). The response rate of the weekly online health surveys dropped by 3% from the pre– to post–cancer diagnosis period (from 89% to 86%). The use rate of the scale was 28% throughout the monitoring period, and there was a slight increase in the use rate after cancer diagnosis across the participants. The median number of days per week per participant when the weight was recorded was 2.7 throughout the monitoring period. The medication pillbox use rate was 53% throughout the monitoring period, and the use rate dropped by 21% from the pre– to post–cancer diagnosis period (from 68% to 47%). The median number of days per week or per participant when a pillbox opening was recorded was 5.2 throughout the monitoring period.

**Table 2:** Measurement of use and response rates across 1 year before and after cancer diagnosis^a^.

	Data points/participant, mean (SD)				Use and response rate (%), mean		
	Total monitoring	Before diagnosis	After diagnosis	Total monitoring		Before diagnosis	After diagnosis
Actigraphy data (d)	336 (125)	210 (88)	126 (93)	64		69	56
Online health survey data (wk)	67 (15)	39 (12)	28 (20)	88		89	86
Scale (d)	143 (85)	70 (59)	73 (72)^b^	28		22	27^c^
Electronic pillbox (d)	221 (128)^c^	118 (91)^b^	102 (75)^b^	53		68^c^	47^c^

^a^Data collected after the COVID-19 pandemic were not included in the analysis.

^b^Data from only 8 participants were used.

^c^Data from only 9 participants were used.

### Physical Activity

**Figure 1:**
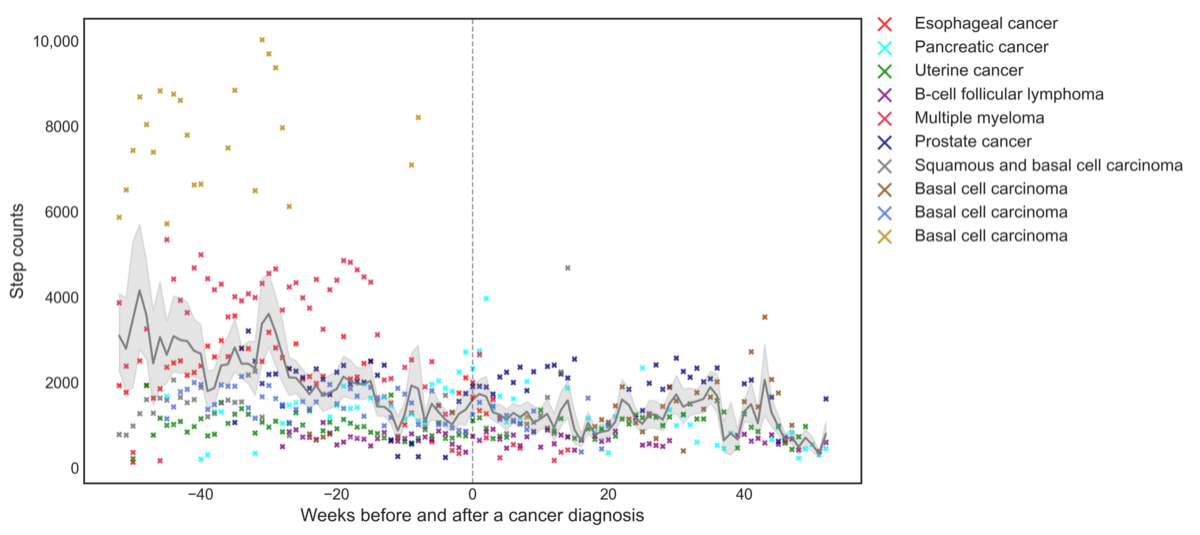
Physical activity 1 year before and after cancer diagnosis (N=10). Weekly average step count (black line) and 95% CI (gray shading) for the cohort are shown for the 1 year before and following a self-report cancer diagnosis using data from the actigraphy watch. Weekly average step counts for each individual are shown by colored dots. Date of reported cancer diagnosis is set at week 0.

Longitudinal linear mixed-effects models revealed that the trajectories of mean daily step counts and step count variability during the precancer diagnosis period was significantly different from that during the post–cancer diagnosis period (precancer diagnosis: β=–1.65; *P*<.001; postcancer diagnosis: β=.003; *P*=.004). Figure 1 shows that there was a steeper decline in step counts over the year preceding the cancer diagnosis compared with a flat slope observed 1 year after the cancer diagnosis. There was a gradual increase in step count variability over the year preceding the cancer diagnosis compared with the variability observed 1 year after the cancer diagnosis. The step count changes were notably different depending on the cancer diagnosis. The participant with esophageal cancer had the most drastic decline in step counts (from 3584.3 to 855.9 step counts) from the pre- to postdiagnosis period, followed by the participant with basal cell carcinoma (from 9521 to 7335 step counts; Table 1). Some participants had increased step counts from the pre- to postdiagnosis period, including those with pancreatic cancer (from 1491 to 1800 step counts) and squamous cell carcinoma (from 733 to 1310 step counts; Table 1).

### Weight

**Figure 2:**
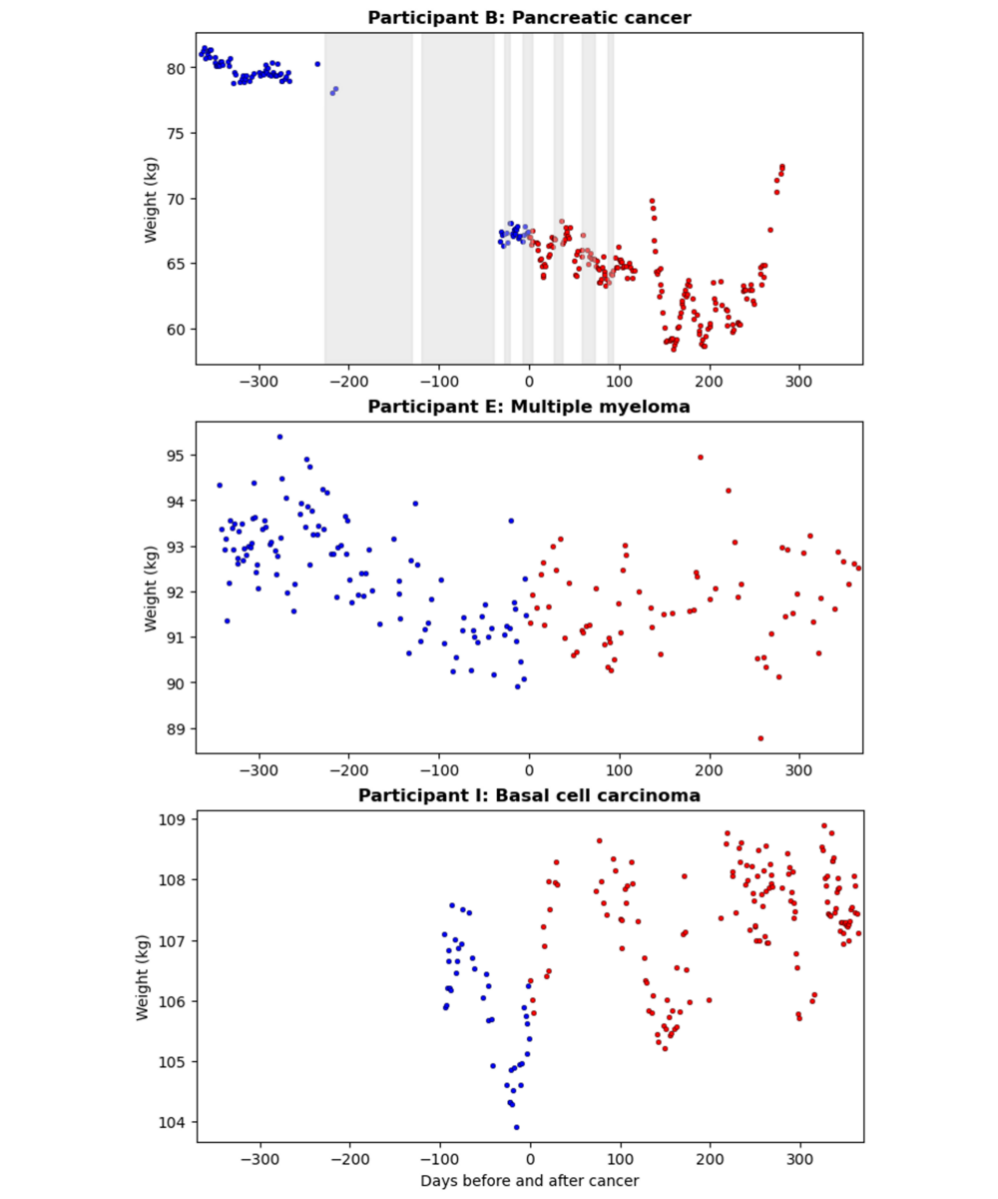
Case examples of weight changes. Daily weight in kilograms from the digital scale is shown for an individual with pancreatic cancer (participant B, top panel); multiple myeloma (participant E, middle panel); and basal cell carcinoma (participant I, bottom panel). Date of reported cancer diagnosis is set at day 0. Blue dots indicate daily weight before the diagnosis; red dots represent daily weight recorded after diagnosis. Vertical gray shading (top panel) indicates days away from home overnight reported via weekly online health survey.

The trajectory of weight differed before and after cancer diagnosis (β=−.01; *P*<.001). There was a steeper weight loss over the year preceding the cancer diagnosis compared with 1 year after the cancer diagnosis when the average weight trajectory remained flat. Table 1 shows the average weights before and after diagnosis. A total of 6 (75%) out of 8 participants lost weight from the pre- to postdiagnosis period on average (2 participants did not have weight data after the diagnosis). The most profound weight loss was found in the participant with pancreatic cancer (12.5 kg), followed by the participant with basal cell carcinoma (4.7 kg) and the participant with uterine cancer (3.8 kg). Figure 2 shows the patterns of weight fluctuations in 3 participants. The participant with pancreatic cancer had a steep decline in weight before the diagnosis, which was followed by an up-and-down weight pattern after the diagnosis. In contrast, the participant with multiple myeloma had a more gradual and steady downward weight loss pattern over time. The participant with basal cell carcinoma had the lowest weight immediately before diagnosis.

### Medication-Taking Behavior

**Figure 3:**
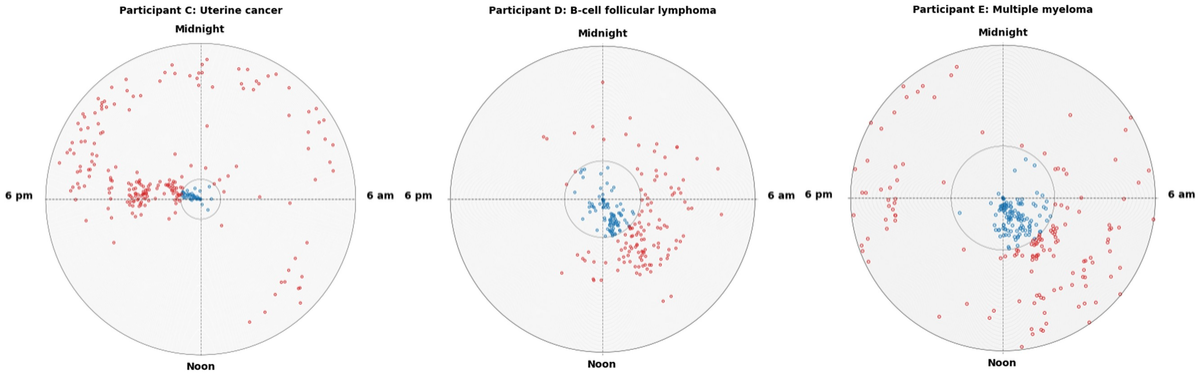
Case examples of medication-taking behavior. Clock time of the first time any pillbox door was opened during each day on the electronic pillbox is shown for an individual with uterine cancer (participant C, left panel); B-cell follicular lymphoma (participant D, middle panel); and multiple myeloma (participant E, right panel). Each dot represents the first time any pillbox door was opened during each day, and the position of each dot around the circle indicates the time of day the pillbox door was opened, represented in 24-hour time; calendar time is read from the center of the grid to the edge of the grid. Date of the reported cancer diagnosis is indicated by the inner concentric circle. Blue dots indicate daily medication taking before the diagnosis; red dots after the diagnosis.

The trajectory of the number of pillbox door openings during the prediagnosis period was significantly different from that during the postdiagnosis period (β=.007; *P*<.001), suggesting that the number of pills that the participants were taking significantly changed over time. There was a gradual increase in the pillbox door openings after the diagnosis compared with a downward pattern during the prediagnosis period. The participants displayed visually and quantitatively distinguishable differences in the time of day when they took their medication and the consistency of this timing, indicating a fluctuation in medication adherence behavior throughout the course of their cancer treatment. Table 1 shows the average number of pillbox door openings and the time of the day of pillbox door opening before and after the diagnosis. Two participants had a greater increase in the pillbox openings per day than the others: 1 participant with multiple myeloma (0.5 openings) and 1 with uterine cancer (1.2 openings). On average, the time of the day of pillbox door opening did not change from the pre- to postdiagnosis period across participants. As shown in Figure 3, the 3 case examples show scatter plots with a broader spread after diagnosis, illustrating changes in medication-taking behavior after the cancer diagnosis. For example, before the diagnosis, an individual with uterine cancer (participant C) typically took medication at around 6 PM, and after the diagnosis, the participant took medications at a variety of times spread across evenings and mornings.

### Pain Severity

**Figure 4:**
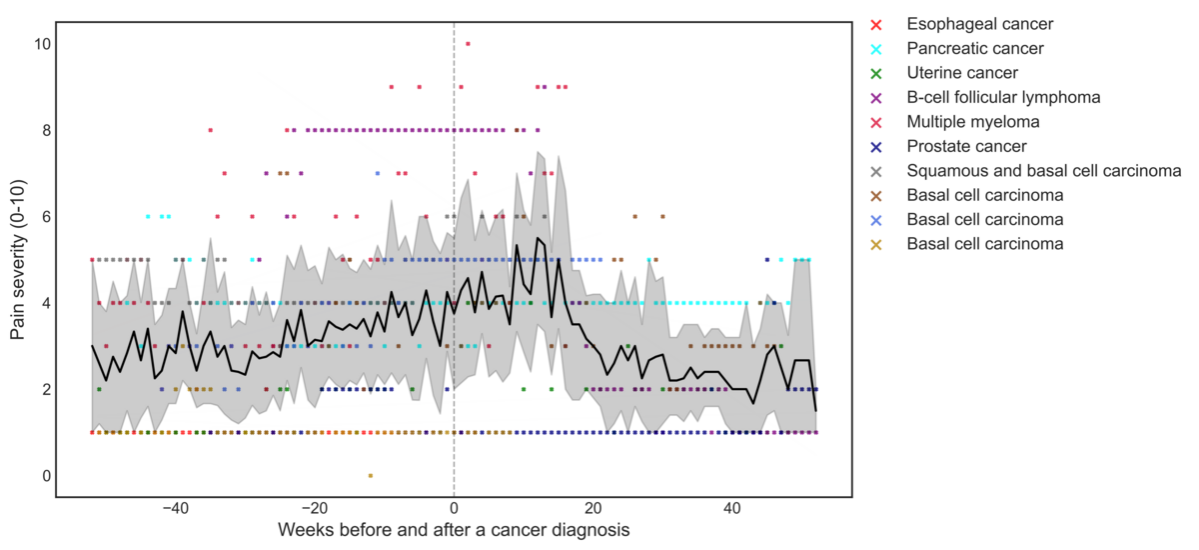
Pain severity 1 year before and after cancer diagnosis (N=10). Weekly average pain severity (black line) and 95% CI (gray shading) for the cohort are shown for the 1 year before and following the self-reported cancer diagnosis. The weekly average pain severity for each individual is shown by colored dots. Date of the reported cancer diagnosis is set at week 0.

The trajectory of weekly pain severity during the prediagnosis period was significantly different from that during the postdiagnosis period (β=.06; *P*<.001). Over the year preceding the cancer diagnosis, there was a gradual increase in pain severity compared with a downward trajectory during the postdiagnosis period. The 2 participants with esophageal and pancreatic cancers had worsened pain severity from the pre- to postdiagnosis period on average (2.2 and 1.5 points higher, respectively). In contrast, the participant with uterine cancer had reduced pain severity from the pre- to postdiagnosis period (4.1 points lower; Table 1). Notably, the group’s average pain severity continued to increase until the 15th week after diagnosis (Figure 4).

### Health Events and Mood

Generalized linear mixed models revealed that the trajectory of the frequency of hospitalization or ER visits during the prediagnosis period was different from that during the postdiagnosis period (odds ratio 1.08, 95% CI 1.01-1.16; *P*=.03). Over the year preceding the cancer diagnosis, there was a gradual increase in the number of hospitalization or ER visits, whereas after diagnosis, the number of hospitalization or ER visits did not increase or decrease. Of all the weeks (n=24 wk) when hospitalization or ER visits were reported, 25% (n=6) were cancer-related visits.

Generalized linear mixed models revealed that the trajectory of the frequency of being away from home during the prediagnosis period was different from that during the postdiagnosis period (odds ratio 1.05, 95% CI 1.01-1.08; *P*=.01). Over the year preceding the cancer diagnosis, there was a gradual increase in the number of days away from home overnight, whereas after diagnosis, the number of days away from home overnight did not increase or decrease. Of all the weeks (n=123 wk) when the participants reported being away from home overnight, 5.7% (n=7) were medical or cancer-related issues.

**Figure 5:**
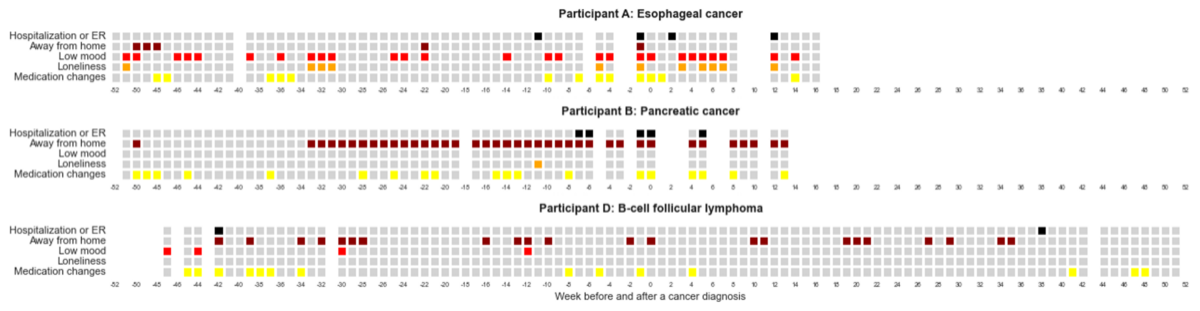
Case examples of weekly health events and mood. Events from the weekly online health survey are shown for an individual with esophageal cancer (participant A, top panel); pancreatic cancer (participant B, middle panel); and B-cell follicular lymphoma (participant D, bottom panel). Each column represents 1 week; date of the reported cancer diagnosis is set at week 0. Gray boxes indicate that no event was reported for that week; colored boxes indicate that an event was reported (black, hospitalization or emergency room [ER] visit; brown, time away from home overnight; red, low mood; orange, loneliness; yellow, medication changes). White columns indicate missing data or weeks of data censored due to death or the COVID-19 pandemic.

There were no differences between the pre– and post–cancer diagnosis periods in terms of low mood (*P*=.60), loneliness (*P*=.22), self-reported medication changes (*P*=.08), or self-reported new medication or dosage increases (*P*=.07). Examples of weekly self-reported events are presented in Figure 5.

**Figure 6:**
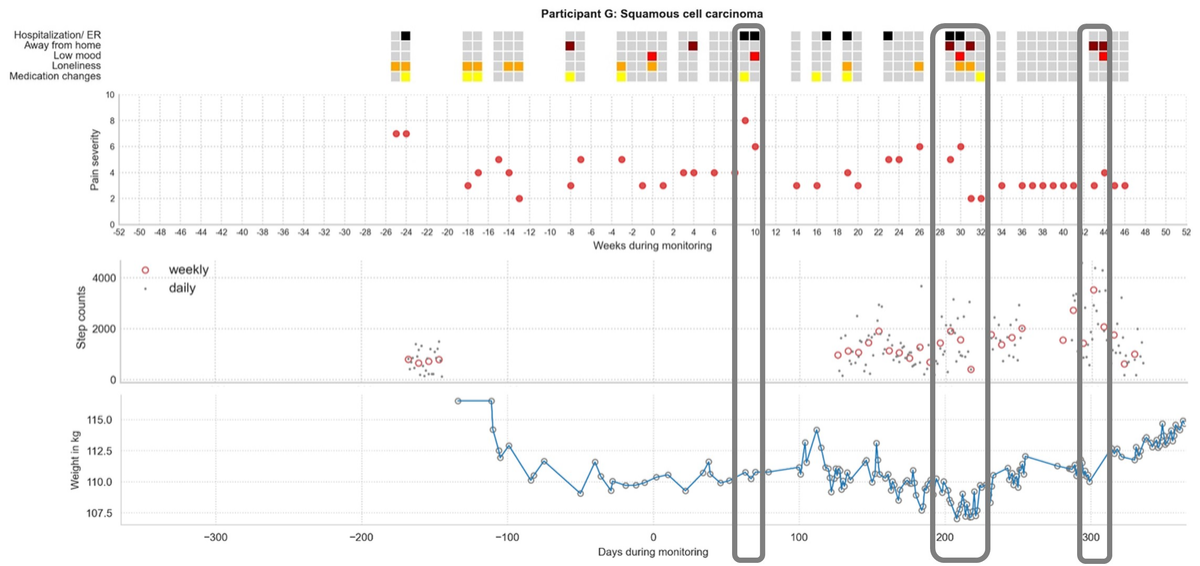
Combined illustration of multiple passive monitoring data streams from a single case before and after the cancer diagnosis. Events from the weekly online health survey (top panel: black, hospitalization or emergency room [ER] visit; brown, time away from home overnight; red, low mood; orange, loneliness; yellow, medication changes); weekly average pain severity from the weekly online survey (second panel: red dot, average weekly pain severity); daily physical activity from the actigraphy watch (third panel: gray dot, daily step count; red open circle, 7-day average step count); and daily weight in kilograms from the digital scale (bottom panel: gray open circle, daily weight) from an individual with squamous cell carcinoma (participant G). Date of the reported cancer diagnosis is set at day or week 0. Coincident events of interest from multiple data streams are indicated by the vertical gray rectangles. Left rectangle: at approximately day 60 after the diagnosis, the participant reported ER visits and was admitted to the hospital. The reason for hospital admission was related to bradycardia. There was elevated pain severity and low mood reported during this period. Middle rectangle: at approximately day 200 after the diagnosis, the participant reported new ER visits and was admitted to the hospital. The reason for hospital admission was related to atrial fibrillation. Pain severity was relatively high, and low mood and loneliness were concurrently reported. Also, average weekly step count transiently declined within the highlighted period, and the participant’s weight dropped to the lowest range observed across the monitored period. Right rectangle: at approximately day 300 after the diagnosis, the participant was away from home overnight for 2 weeks. During this period, there were increased step counts collected from the actigraphy watch and an increase in weight, and low mood was reported. Note: participant G experienced discomfort when wearing an actigraphy watch because of the large wrist. A specially made leather wristband was provided to the participant, but it did not reduce the discomfort; overall, there were scarce watch data to present.

Figure 6 depicts an integrated view of the changes in multiple domains of health over time for a single individual. In this case, an individual with squamous cell carcinoma (participant G) had simultaneous data collection of self-reported health events, mood, pain severity, body weight, and physical activity before and after the cancer diagnosis. During the monitoring period, we identified co-occurring events of interest across multiple health domains. For example, participant G reported a hospitalization event related to atrial fibrillation on day 200 after the diagnosis, which coincided with elevated pain severity, low mood, loneliness, a transient decline in physical activity, and participant G’s lowest weight during the monitoring period. These results illustrate how integrating multiple high-frequency data streams can provide a more holistic picture of an individual’s health over time than episodic assessments or any single high-frequency data source.

## Discussion

### Principal Findings

In this proof-of-concept study, we demonstrated the ability of a multidomain, technology-agnostic assessment platform to detect meaningful intraindividual changes in older adults 1 year before and 1 year after a cancer diagnosis. We provided examples of 4 measurement approaches (wearable devices [actigraphy watch], physiological monitoring devices [electronic scale], electronic pillboxes, and online surveys) that are integrated into a single platform. These largely passive assessments impose a minimal burden on older adults who are newly diagnosed with cancer, as shown by the substantial use and response rates in this cohort. Compared with conventional periodic assessments, the platform collected a higher frequency of data on daily activities, mobility, health events, and symptoms, and these measures have been validated in a larger cohort of older adults [10-12,15,17-24]. Although our results are limited to a case series of 10 older adults with cancer of various types and severities, they suggest that an integrated platform provides a methodological pathway for surveilling long-term behavioral, health, and clinical outcomes for older cancer survivors who will live with cancer for many years.

Using a largely passive multifunctional assessment approach, such as the one described here, may improve the assessment of treatment toxicity and tolerance on multiple dimensions of quality of life that are important to older patients. Older cancer survivors face unique health problems compared with their younger peers in terms of comorbidities and treatment tolerability. Cancer treatment can accelerate the development of frailty [25,26] and cognitive impairment [27], while older adults experience polypharmacy and drug interactions with chemotherapy [28] that add to the complexity of their care. A multicenter study of older cancer survivors documented that half of the participants experienced at least one grade 3 to 5 chemotherapy-related toxicity [29]. An in-depth understanding of the unique impact of cancer and cancer treatment on health and functioning in older patients is limited because they are poorly represented in clinical trials, and we lack longitudinal studies following older patients from diagnosis to the end of life. The CART initiative was explicitly funded as a platform that is widely used by the national research community to facilitate the investigation of the impact of multiple diseases on key dimensions of daily life to better identify opportunities to improve the health span and independent living of older adults. In the context of cancer, symptoms collected from a passive home-based assessment platform can provide more timely detection of toxicity (eg, mobility or weight changes) in patients receiving cancer therapy or participating in clinical trials. In addition, the high response rate of weekly online surveys in this study (which typically take <5 min to complete) supports the feasibility of collecting weekly self-reported data, including treatment-related symptoms [13]. Mood changes, pain levels, medication adherence, and related salient data are not generally reported until a crisis occurs; therefore, regular online reporting provides a proactive means to prevent poor outcomes. Applied to the development of cancer therapeutics, this integrated approach can transform the current episodic and subjective monitoring of adverse events into much more timely, real-world, and precise examinations of patient functioning across multiple domains that contribute to quality of life. These data may serve as an essential adjunct to assessing the therapeutic toxicities and physical decline and, in particular, to understand the unique response and tolerance to treatment in older patients. Moreover, clinicians can translate these data to respond in real time to patients to adjust therapies and to optimize supportive care strategies that can be rapidly deployed to minimize cancer’s overall morbidity and improve long-term outcomes.

An at-home, technology-based assessment platform could also potentially reduce health care disparities for older adults with cancer, such as those living in rural areas or those with limited ability to travel to a major cancer center. Patients in rural areas, particularly those with low socioeconomic status, experience various barriers to care (eg, transportation and distance to specialty care). This platform could be used to obtain frequent assessments of symptoms, mobility, and important health behaviors that could be electronically reported to clinicians in near real time [30]. Integrating passive monitoring into cancer survivorship research, particularly for older patients with a complex medical history and those with limited access, could advance care and ultimately improve quality of life.

In addition to integrating into cancer therapeutic trials and addressing health disparities, a passive monitoring assessment platform offers additional benefits. The design is purposely flexible to expand the scope of other possible measures, such as sleep by using electronic bed mats; cognitive function by monitoring computer use, with software installed on home devices, or by monitoring driving function with a sensor deployed to the data port of a vehicle [12,21,22,31]; or socialization by monitoring time spent outside home using motion and door contact sensors installed in a participant’s home [32]. In addition, the advantage of high-frequency data collected by passive monitoring approaches is the flexibility to adjust time granularity. Researchers can condense time-stamped data into a coarser time granularity (interdaily, weekly, or monthly) to monitor their outcomes of interest. Studies have found that frequent assessment of symptoms using electronic reporting can improve survival rates in patients with metastatic disease [33,34]. For patients in the early phases of adjuvant therapies, particularly in the experimental setting, intradaily or daily data could help monitor acute responses to treatment and toxicity. For cancer survivors who are on long-term maintenance therapy or who have completed treatment altogether, weekly or monthly data can be used to not only track long-term mental and physical health but might also be used to identify the trajectory of onset for long-term and late effects of treatment. In addition, time-stamped high-frequency data might provide insight into the causal relationships between behaviors and symptoms or side effects that cannot be achieved with lower-frequency data that are typically obtained in observational cohort studies. For example, the time of a day when a patient opens the pillbox compartment door can be correlated with step counts within the next few hours. Daily and weekly weights can be linked to hospitalization and ER visits. These pieces of critical information reveal patterns of symptoms and side effects that can inform when and how to administer treatment [35].

### Limitations

As this is a retrospective study that did not initially focus on cancer, we did not have medical records regarding the exact diagnosis date, cancer stages, invasive or noninvasive malignancies, or type of treatments to interpret individual data patterns. Future studies would benefit from prospective surveillance with access to electronic medical records. Our analysis included heterogeneous types of cancer, and some cancers are known to be less aggressive and require less treatment (eg, basal cell carcinoma) than others (eg, pancreatic cancer), limiting our ability to interpret data across the sample. In addition, common cancers including breast and colorectal cancers were missing from the small sample. However, these data suggest that changes in behaviors, symptoms, and health across survivorship that may precede and follow a specific health event during that time, such as a start or change in therapy or cancer recurrence, could be readily captured with little added burden to individual patients. Conceptually, the aggregated cancer data suggest the feasibility of population-level surveillance, in addition to informing individual diagnosis-specific case management.

### Future Directions

Beyond its utility in unobtrusively monitoring the health and behavior of older adults with cancer, this passive monitoring platform also offers a tool to monitor the health of their caregivers. Caregivers of older cancer survivors play a vital role in monitoring medication adherence, symptoms, and medical appointments. Caregivers often experience low moods and loneliness throughout the cancer journey of the cancer survivor. Although this study did not examine the dyadic interactions of mood and physical activities among older dyads, this research area is of interest for maintaining the well-being of both cancer survivors and their caregivers. Finally, the capability of this platform to offer multiple data streams will allow researchers to build multivariable predictive models to predict early cancer onset and recurrence, treatment toxicity, quality of life outcomes, and longevity.

### Conclusions

In this proof-of-concept study using a secondary analysis of an established cohort of older adults, we demonstrated the utility of a multidomain assessment platform to detect intraindividual changes associated with distinctive trajectories in older persons with cancer. Continuous passive monitoring of function overcomes many limitations of self-reported, clinic-based, and infrequent measures in longitudinal studies, providing an opportunity to follow multiple dimensions of function across the entire trajectory of cancer survivors (ie, from diagnosis through to the end of life). Using home-based digital technology to measure key functional domains (ie, mobility, cognitive and behavioral health, and socialization) could provide a comprehensive understanding of the changes that older cancer survivors experience as they occur in real life. By 2040, the proportion of cancer survivors who are aged >65 years will rise to 73%, and most will survive for ≥5 years [36]. Within just 5 years, the number of older survivors in the United States alone is projected to reach 14 million. Our proof-of-concept study supports the integration and application of digital technologies to support the care of older cancer survivors.

## Abbreviations

CART: Collaborative Aging Research Using Technology
ER: emergency room

## Data Availability

Data will be available upon request.
